# Evaluating Partnerships for Practice Change in the Prevention, Identification, and Treatment of Fetal Alcohol Spectrum Disorders

**DOI:** 10.24966/AAD-7276/100077

**Published:** 2022-01-12

**Authors:** Saloni Sapru, Patricia Green, Mary Kate Weber, Melanie Chansky, Simani Price

**Affiliations:** 1 Westat, 1600 Research Boulevard, Rockville, Maryland, USA; 2 Centers for Disease Control and Prevention, 4770 Buford Hwy, Atlanta, Georgia, USA

**Keywords:** Alcohol use during pregnancy, Collaboration, Fetal alcohol spectrum disorders

## Abstract

**Background:**

In 2014, the Centers for Disease Control and Prevention funded a four-year partnership effort between university and health care professional associations (HCPAs) to reach health care providers (HCPs) nationally in six health disciplines and engage them to adopt evidence-based practices for the prevention, identification, and treatment of fetal alcohol spectrum disorders (FASDs). The aim of this project was to evaluate partnerships for their (1) structure and formation, (2) collaboration process, and (3) outcomes with regard to resources and strategies developed for FASD prevention and management.

**Methods:**

We used quantitative and qualitative data from quarterly progress reports, a semi-annual collaboration survey, and annual interviews with each discipline’s members.

**Results:**

Partnerships in each discipline varied in the number of members and organizations, expertise in the discipline, and access to HCPs. Assigned partnerships with limited researchers’ expertise in the discipline or the inability of HCPAs to reach priority audiences created challenges in the development and dissemination of resources. Two partnerships showed challenges in the collaboration process regarding understanding respective responsibilities, sharing similar ideas, and resolving disagreements despite efforts at facilitated discussion. Messaging and resource dissemination by HCPAs and the use of provider champions developed through HCPAs’ national network emerged as promising approaches to engage HCPs.

**Conclusion:**

Circumstances under which partnerships are formed can facilitate or challenge collaboration and outcome efforts. Discipline-specific partnerships between researchers and HCPAs provide a model for evidence-based resources to be developed and disseminated widely for adoption by HCPs in their practice.

## Background

Health care providers (HCPs) play a critical role in preventive care such as by employing alcohol screening and brief intervention (SBI) in routine practice to identify risky alcohol use among patients. However, reaching and engaging HCPs to apply such evidence-based practices like alcohol SBI in routine clinical care requires collaboration and coordination between various partners. Effective partnerships can help translate evidence-based research into practice [1]. Dissemination and implementation science examines how scientific evidence is adopted, implemented, and sustained in community or clinical settings [2,3]. A collaborative model of researchers, practitioners, or other stakeholders can improve the likelihood that evidence is both implemented and sustained in everyday practice [4,5]. Collectively addressing public health challenges can ensure that advances in health science become standards for care across populations and health care settings [6].

Partnerships create synergy, allowing individual organizations to combine human and material resources to accomplish objectives which they could not alone. Partnerships, however, need skills and expertise as well as resources, such as connections to priority populations, decision-makers, or key opinion leaders [7]. Finally, high levels of synergy also depend on relationships based on trust, ability to manage conflict, and power differentials [8,9]. In 2014, the Centers for Disease Control and Prevention (CDC) funded a four-year collaborative effort between university-based “Practice and Implementation Centers” (PICs) and “Partners” representing national health care professional associations (HCPAs). The PIC-Partner effort was to center on practice change at the systems level for the prevention, identification, and treatment of fetal alcohol spectrum disorders (FASDs), which are lifelong physical, behavioral, and intellectual problems and are preventable if an individual is not exposed to alcohol before birth [10].

CDC’s PICs and Partners collaboration was conceptualized such that PICs would focus on the development of training materials and resources designed to promote evidence-based, clinical practices such as alcohol SBI, which has shown to be effective at reducing excessive alcohol use [11]. The Partners were to assist the PICs in reaching members of HCPAs with opportunities for messaging, education, and training. HCPAs typically serve the interest of their membership and help to advance medical knowledge, playing a principal role in setting standards of performance, shaping practice, and providing education and leadership. They can be influential in facilitating change at the systems’ level by promoting practices, policies, and interventions.

The PIC-Partner collaboration was to occur in discipline-specific workgroups (DSWs) and each DSW was to target providers practicing in primary care settings in one of six health disciplines of family medicine, medical assisting, nursing, obstetrics/gynecology, pediatrics and social work. Each of the six health disciplines plays a different role in the prevention, identification and treatment of FASDs. For example, pediatricians are typically involved with the identification and diagnosis of children with FASDs; obstetricians and gynecologists focus on prevention of alcohol use during pregnancy with their patients; social workers often provide behavioral health counseling and facilitate referrals; family medicine practitioners take an integrated care approach to prevention and patient care; medical assistants have a scope of practice set by physicians and varies by state; and nursing providers’ continuum of care can range from prevention to identification and treatment. An understanding of the provider group and how each one operates within the health system was critical for each discipline. The national HCPAs provide such understanding and can help with dissemination of continuing education, training, guidelines and technical updates.

## Aim

The aim of this paper is to evaluate partnerships within DSWs with a focus on (1) their structure and formation; (2) the collaboration process; and (3) outcomes of collaborations resulting in resources and strategies developed.

## Methods

Quantitative and qualitative data from different sources were collected to examine DSWs structure, collaborative processes, and outcomes. Table 1 lays out the data collection and analytic methods for the evaluation.

## Results

### DSW partnership structure and formation

CDC’s initial vision was for a PIC to collaborate with a national HCPA (called a Partner) in a DSW and reach providers of its health discipline. However, based on the applicants eventually funded for the initiative, DSWs resulted in different numbers of partnering organizations (ranging from 2 to 6) and combinations of expertise and ability to reach priority audiences (Table 2). The number of active members within each DSW ranged from 5 to 11 over the four years of the collaborative effort. The DSWs, de-identified here to maintain anonymity, are described as A, B, C, D, E, and F.

The groups worked as best as they could with their paired organizations. The evaluation identified some facilitators and challenges to their collaborations. The PICs’ content expertise and Partners’ access to priority audiences facilitated collaborative activities. For example, DSW B comprised two PICs with abundant expertise in the discipline. Further, the paired Partner was a funded, national HCPA with the ability to leverage its association’s experts and dissemination channels. With these resources, this DSW was able to form quickly, develop an action plan, clarify responsibilities, schedule regular meetings and form task-based sub-groups. During an interview, one member described the partnership experience as a “learning community,” with flexibility and adaptability as being critical for the DSW’s success. This member added, “You’ve got to kind of put aside any ego or personal agenda to be able to make the collaboration work.” Each person in the DSW volunteered for tasks and was willing to step in to help in this DSW - “everyone is willing to step back and let someone who has a strength in a certain area step up and lead a particular task,” explained this DSW participant. In contrast, members of DSWs C and E reported delays in activities either due to the PIC’s limited disciplinary expertise or lack of a Partner that could reach the priority audience. To reach their audiences for dissemination, some PICs and Partners had to sub-contract with national HCPAs not funded by CDC, resulting in fewer financial resources and a more limited scope of work.

### DSW collaboration process

**Figure 1:**
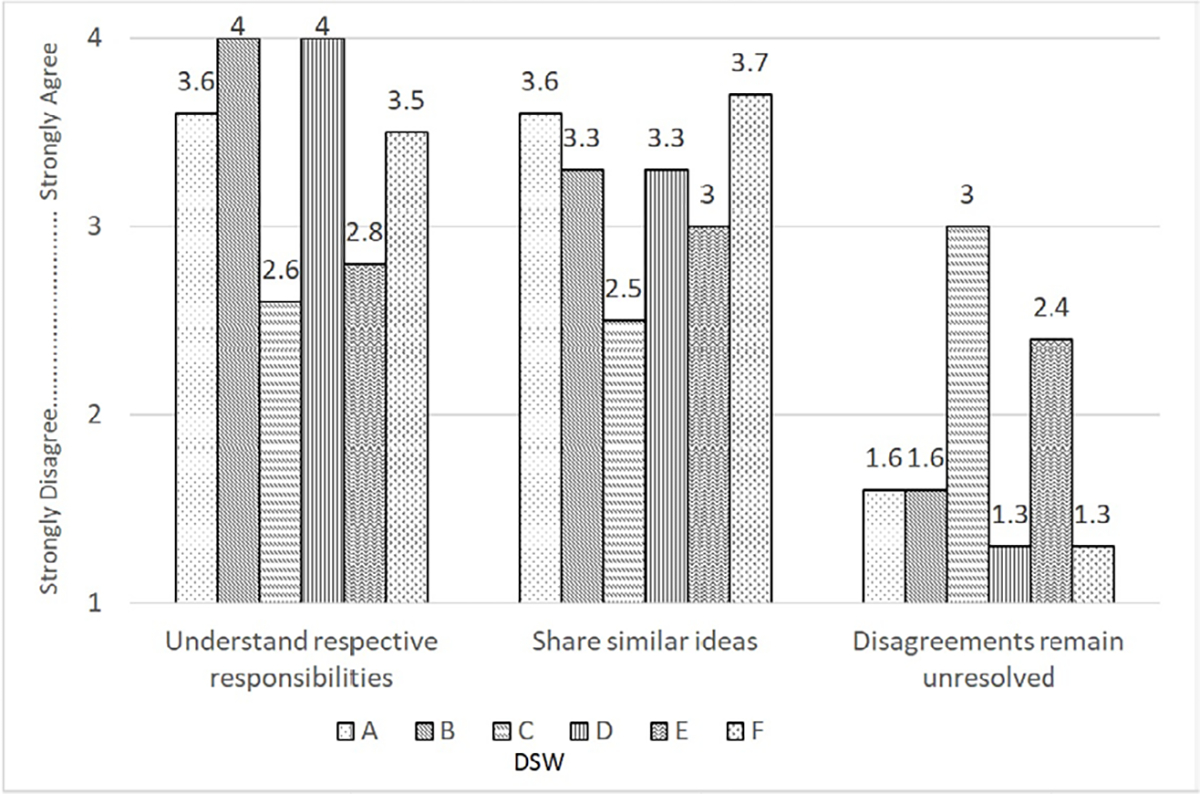
Average DSW scores in the last six months on the collaboration survey. **Abbreviation:** DSW, discipline-specific workgroup

The results of the semi-annual survey [12] used to assess collaboration processes within the DSWs were similar within each DSW over the course of the project. The results of the average DSW scores during the last six months of collaboration indicated that DSWs A, B, D and F had relatively high agreement scores (i.e., more than 3 on a scale of 1 to 4, progressing from strongly disagree to strongly agree) on understanding responsibilities, accommodating schedules, sharing ideas, discussing individual issues, cooperating on new plans, asking opinions, anticipating the need for help and passing important information. Their average scores were also very low (i.e., less than 2) on the item “disagreements remain unresolved,” indicating successful resolution of conflicts, if encountered. In contrast, DSWs C and E indicated lower agreement scores on almost all measures and higher agreement scores for the measure “disagreements remain unresolved.” Interview findings similarly indicated collaboration challenges for DSWs C and E. By the second year of the program, members of one of the two PICs of these DSWs requested to discontinue working with their Partner. Members of the other DSW (also facing collaboration challenges) indicated they did not need the Partner assigned to them. To help these two DSWs work together, CDC offered facilitated discussion. Interview data indicated that this discussion resulted in improvements in setting collective goals, schedules and operational procedures. However, in other domains, facilitated discussion did not substantially improve collaborative functioning. For example, challenges seemed to persist as indicated by average scores on items “understand respective responsibilities,” “share similar ideas,” and “disagreements remain unresolved” on the collaboration survey even in the last six months of the partnerships (Figure 1).

### Outcomes of DSW collaborations

Position/policy statements by HCPAs provide their members a rationale to support a particular viewpoint on a health issue. Similarly, training materials and other resources provide education and guidance. Given the different foci of each discipline, the DSWs developed a range of materials tailored to their respective priority audience which are available through CDC’s FASD Training and Resources website created for this program (see www.cdc.gov/FASDtraining).

CDC-funded Partners that were national HCPAs from DSWs B and E, facilitated systems-level change through innovative strategies. For example, one identified content experts to develop and pilot an implementation guide to assist providers to screen for Prenatal Alcohol Exposure (PAE). The other HCPA developed a speakers’ bureau to help project champions navigate and schedule speaking events in over 200 residency programs across the country. These Partners from DSW B and E also developed a national cadre of champions through their national HCPA’s network of districts/regions to influence practice change among their peers. PICs also initiated champions’ activities ranging from hiring representatives of national HCPAs to developing position statements/conference abstracts or conduct membership surveys; recruiting individuals for trainings-of-trainers; training office-based providers who would facilitate alcohol SBI in their clinical practices; and identifying “student-based,” “executive/leadership-based,” and “content-based” champions. The PIC-based champions’ initiatives showed little promise of sustainability, primarily because they were a loose network of individuals and keeping them engaged was challenging. One PIC member noted: “we haven’t found that our champions take things and run with them - writing an abstract, submitting an abstract for a conference - that’s not stuff they will do on their own.”

Other systems-level efforts included an HCPA approving Maintenance Of Certification (MOC) for training courses completed by providers and eight position/policy statements or guidelines promoted and disseminated by all HCPAs except one.

## Discussion

The evaluation of the partnership model promoted by CDC between researchers and HCPAs highlights elements of the structure of discipline-specific groups, the collaboration process, and outcomes that have implications for engaging HCPs to adopt evidence-based practices in patient care. Assigned collaborations with limited expertise in the discipline or the inability to reach priority audiences created partnership challenges. Furthermore, a team can face challenges if members are unable to cooperate, coordinate, and communicate well together [8], as some results of the collaboration survey revealed.

The development of training materials and resources on preventing alcohol use during pregnancy was a significant outcome of partnerships fostered in this project, all of which are publicly available on-line. National HCPAs facilitated wide-scale dissemination of resources and messaging. Their dissemination of position/policy statements and guidelines are examples of practice change efforts that are important to influence health policy development and improve health care standards at the systems level.

Using champions to influence peer providers was another promising strategy. Champions can promote uptake of interventions [13]. They are also key in sustaining interventions such as Screening, Brief Intervention, and Referral to Treatment (SBIRT) when federally supported funding ceases, and can help address stigma in medical settings and the larger community when patients do not want SBIRT services documented in their medical records because of their association with substance abuse [14]. The evaluation findings showed that with adequate resources, national HCPAs could develop effective national-level champions’ initiatives through their membership network. Champion efforts by the national HCPAs were directed primarily at raising awareness about FASDs and alcohol use during pregnancy among peers. A focus on the implementation of evidence-based interventions by HCPs would be the next step for this promising strategy.

## Limitations

The evaluation was completed at the end of four years, but PICs and Partners continued with their project activities for several months after this time through no-cost extension agreements with the CDC. Terminating the evaluation prior to the completion of the programs was a limitation, as longer-term outcomes could not be assessed.

### Implications for practice

Discipline-specific partnerships between university-based researchers and national health care professional associations provide a model for evidence-based resources to be developed and disseminated widely for adoption by health care providers in their practice. Lessons learned from this initiative informed a subsequent CDC funding cycle in 2018 in which grantees use national HCPA champion networks, continue dissemination of resources, and facilitate implementation of alcohol SBI protocols in primary care clinics using system-level approaches, such as integration of protocols in electronic health records.

## Figures and Tables

**Table 1:** Data collection sources, methods and analysis.

	Data Source		
	DSW progress reports	Web-based collaboration survey*	Interviews with active DSW members
Frequency of data collection	Quarterly	Semi-annual	Annual
Data collection method	Pre-developed templates completed by DSWs on update of activities, including: - Quantitative program data (e.g., on priority audience reach, trainings, resources disseminated) - Description of DSW strategies and challenges	10-item questionnaire with Likert scale responses (1=strongly disagree to 4=strongly agree) completed by all active DSW members to measure communication, accommodation, and isolation within the DSW	Telephone or in-person interviews using a semi-structured discussion guide to obtain different perspectives of members within the DSWs. Recorded interviews with participants’ consent and professionally transcribed
Data analysis	- Frequencies (counts) developed of quantitative program data - Qualitative descriptions categorized to identify strategies used and challenges encountered	Used SPSS 25 software for descriptive statistical analysis to obtain average scores for each item for a DSW	Used NVivo 11 software to code transcripts based on scheme developed by two evaluators after reviewing 10% of transcripts and reached 89% agreement for all codes Content analyzed transcripts using deductive categories to identify themes across DSWs

* The survey was adapted from the scale for interprofessional collaboration by Kenazchuk Reeves, Nicholas and Zwarenstein [12].

**Abbreviation:** DSW, discipline-specific workgroup

**Table 2:** Elements of DSW formation.

	DSW					
	A	B	C	D	E	F
At onset PIC had no paired Partner or Partner had no paired PIC	✓			✓	✓	
PIC’s paired Partner was not a national HCPA			✓			✓
HCPA sub-contracted by PIC or Partner	✓		✓	✓		✓
More than one PIC paired with Partner		✓	✓			✓
PIC staff did not have expertise in the health discipline of the HCPA			✓	✓	✓	

**Abbreviations:** DSW, discipline-specific workgroup; PIC, Practice and Implementation Center; HCPA, health care professional association.

## References

[1] Stokols D, Hall KL, Taylor BK, Moser RP (2008) The science of team science: Overview of the field and introduction to the supplement. Am J Prev Med 35: 77–89. 18619407 10.1016/j.amepre.2008.05.002

[2] Bauer MS, Damschroder L, Hagedorn H, Smith J, Kilbourne AM (2015) An introduction to implementation science for the non-specialist. BMC Psychol 3: 32. 26376626 10.1186/s40359-015-0089-9 PMC4573926

[3] Estabrooks PA, Brownson RC, Pronk NP (2018) Dissemination and implementation science for public health professionals: An overview and call to action. Prev Chronic Dis 15: 162. 10.5888/pcd15.180525 PMC6307829 30576272

[4] Feuerstein JL, Olswang LB, Greenslade KJ, Dowden P, Pinder GL, (2018) Implementation research: Embracing practitioners’ views. Journal of Speech Language and Hearing Research 61: 645–657. 10.1044/2017_JSLHR-L-17-0154 PMC6195068 29450483

[5] Olswang LB, Goldstein H (2017) Collaborating on the development and implementation of evidence-based practices: Advancing science and practice. Evidence-Based Communication Assessment and Intervention 11: 61–71.

[6] Glasgow RE, Vinson C, Chambers D, Khoury MJ, Kaplan RM, (2012) National Institutes of Health approaches to dissemination and implementation science: Current and future directions. Am J Public Health 102: 1274–1281. 22594758 10.2105/AJPH.2012.300755 PMC3478005

[7] Lasker RD, Weiss ES, Miller R (2003) Partnership synergy: A practical framework for studying and strengthening the collaborative advantage. Milbank Q 79: 179–205. 10.1111/1468-0009.00203 PMC2751192 11439464

[8] Jones J, Barry MM (2011) Exploring the relationship between synergy and partnership functioning factors in health promotion partnerships. Health Promot Int 26: 408–420. 21330307 10.1093/heapro/dar002

[9] Jones J, Barry MM (2018) Factors influencing trust and mistrust in health promotion partnerships. Glob Health Promot 25: 16–24. 27466248 10.1177/1757975916656364

[10] Riley EP, McGee CL (2005) Fetal alcohol spectrum disorders: An overview with emphasis on changes in brain and behavior. Exp Biol Med (Maywood) 230: 357–365. 15956765 10.1177/15353702-0323006-03

[11] US Preventive Services Task Force, Curry SJ, Krist AH, Owens DK, Barry MJ, (2018) Screening and Behavioral Counseling Interventions to Reduce Unhealthy Alcohol Use in Adolescents and Adults: US Preventive Services Task Force Recommendation Statement. JAMA 320: 1899–1909. 30422199 10.1001/jama.2018.16789

[12] Kenaszchuk C, Reeves S, Nicholas D, Zwarenstein M (2010) Validity and reliability of a multiple-group measurement scale for interprofessional collaboration. BMC Health Serv Res 10: 83. 20353577 10.1186/1472-6963-10-83 PMC2867963

[13] Miech EJ, Rattray NA, Flanagan ME, Damschroder L, Schmid AA, (2018) Inside help: An integrative review of champions in healthcare-related implementation. SAGE Open Med 6: 2050312118773261. 29796266 10.1177/2050312118773261 PMC5960847

[14] Singh M, Gmyrek A, Hernandez A, Damon D, Hayashi S (2017) Sustaining screening, brief intervention and referral to treatment (SBIRT) services in health-care settings. Addiction 112: 92–100. 28074565 10.1111/add.13654

